# Transcriptome Profiling of Two Asparagus Bean (*Vigna unguiculata subsp*. *sesquipedalis*) Cultivars Differing in Chilling Tolerance under Cold Stress

**DOI:** 10.1371/journal.pone.0151105

**Published:** 2016-03-08

**Authors:** Huaqiang Tan, Haitao Huang, Manman Tie, Yi Tang, Yunsong Lai, Huanxiu Li

**Affiliations:** 1 College of Horticulture, Sichuan Agricultural University, Chengdu, Sichuan, China; 2 Mianyang Institute of Agricultural Sciences, Mianyang, Sichuan, China; 3 Dazhou Institute of Agricultural Sciences, Dazhou, Sichuan, China; Iwate University, JAPAN

## Abstract

Cowpea (*V*. *unguiculata* L. Walp.) is an important tropical grain legume. Asparagus bean (*V*. *unguiculata ssp*. *sesquipedialis*) is a distinctive subspecies of cowpea, which is considered one of the top ten Asian vegetables. It can be adapted to a wide range of environmental stimuli such as drought and heat. Nevertheless, it is an extremely cold-sensitive tropical species. Improvement of chilling tolerance in asparagus bean may significantly increase its production and prolong its supply. However, gene regulation and signaling pathways related to cold response in this crop remain unknown. Using Illumina sequencing technology, modification of global gene expression in response to chilling stress in two asparagus bean cultivars—“Dubai bean” and “Ningjiang-3”, which are tolerant and sensitive to chilling, respectively—were investigated. More than 1.8 million clean reads were obtained from each sample. After de novo assembly, 88,869 unigenes were finally generated with a mean length of 635 bp. Of these unigenes, 41,925 (47.18%) had functional annotations when aligned to public protein databases. Further, we identified 3,510 differentially expressed genes (DEGs) in Dubai bean, including 2,103 up-regulated genes and 1,407 down-regulated genes. While in Ningjiang-3, we found 2,868 DEGs, 1,786 of which were increasing and the others were decreasing. 1,744 DEGs were commonly regulated in two cultivars, suggesting that some genes play fundamental roles in asparagus bean during cold stress. Functional classification of the DEGs in two cultivars using Mercator pipeline indicated that RNA, protein, signaling, stress and hormone metabolism were five major groups. In RNA group, analysis of TFs in DREB subfamily showed that ICE1-CBF3-COR cold responsive cascade may also exist in asparagus bean. Our study is the first to provide the transcriptome sequence resource for asparagus bean, which will accelerate breeding cold resistant asparagus bean varieties through genetic engineering, and advance our knowledge of the genes involved in the complex regulatory networks of this plant under cold stress.

## Introduction

Low temperature is one of the major environmental factors limiting growth, development and geographical distribution of plants, and causes significant losses periodically [1, 2]. Stress caused by low temperature can be divided into chilling stress (0–15°C) and freezing stress (<0°C). Most temperate plants, such as spinach and *Arabidopsis*, possess various degree of chilling tolerance and can acquire freezing tolerance after exposure to non-freezing temperatures, a process known as cold acclimation [3]. On the other hand, some plants from tropical and subtropical regions, like rice, maize and tomato, are easily damaged by chilling stress due to the inability for cold acclimation. In *Arabidopsis* and winter cereals, the mechanism of cold acclimation and acquired freezing tolerance has been extensively investigated [4]. In order to adapt to cold stress during acclimation, expression of genes is reprogrammed and metabolism is altered. Cold tolerance is a highly complex trait, a large amount of genes are involved in cold response of plants [5]. 4%–14% of *Arabidopsis* transcriptome is cold responsive [6]. Cold stress triggers membrane rigidification and cytoskeleton reorganization of cells, which is followed by Ca^2+^-influx from both extra- and intra-cellular sources and activation of protein kinase cascades, eventually leading to activation of transcription factors and expression of COR (cold-regulated) genes [7].

Cowpea, *Vigna unguiculata* L. Walp. (2n = 2x = 22), is an important grain legume, which ranks fifth worldwide as a source of plant protein and fiber [8]. Cowpea is presumed to be originated from Africa [9], and the subspecies *sesquipedialis* is one of the main cultivated divisions of cowpea worldwide [10]. It is also called asparagus bean or ‘yard long’ bean, and is mainly cultivated in eastern and southern Asia for production of immature green pods [11]. Current cowpea varieties are sensitive to chilling, and the cool night temperatures of spring would greatly influence its growth and flowering. So far, some progress has been made by Ismail’s group in breeding cowpeas with chilling tolerance during emergence [12]. They proposed that chilling tolerance during emergence is conferred by the presence of a specific dehydrin protein in the seed [13]. Later, they proved the hypothesis that the dehydrin protein confers an increment of chilling tolerance under single nuclear gene inheritance [14, 15].

On the other hand, research on low temperature response in asparagus bean is extremely limited by lack of cold tolerant material. Dubai bean is a variety introduced from Dubai, the United Arab Emirates, by Mianyang Institute of Agricultural Sciences, Mianyang, Sichuan, China. Most asparagus bean cultivars cannot survive in late autumn (November) except Dubai bean, which makes it a cold tolerant variety. However, it bears less pods and its productivity is poor. Therefore, improving cold tolerance of asparagus bean cultivars is possible by crossing, but some unfavorable agronomic traits may be imported as well. Although several major-effect QTLs can be identified and cloned by QTL mapping, this is hard to achieve due to the fact that cowpea is an entirely self-pollinated crop [16], the success of artificial pollination has been reported to be low, and that the preparation of mapping populations can take years, and map-based cloning is tedious, hampering the quick identification of candidate genes [17]. To improve cold tolerance without obstacles mentioned above, developing transgenic plants is a fast and effective biotechnological tool. Moreover, with the advancement of RNA-Seq, which is a recently developed approach to transcriptome profiling that uses deep-sequencing technologies [18], it allows us to rapidly analyze cold responsive genes of plants, and use “reverse genetics” strategy to identify candidate genes, thus providing available gene resources for molecular breeding.

Comparative analyses have shown that there are some conserved cold-regulated genes that have fundamental roles in plant growth and development during cold stress [19]. However, there are also considerable differences in the sets of genes that comprised the low temperature transcriptomes between different species [19–21] and dissimilar genotypes [22–24]. In order to explore gene regulation and signaling pathways in asparagus bean when subjected to cold stress, and analyze the differences between two varieties, we used RNA-seq to investigate gene expression patterns between Dubai bean (cold tolerant) and Ningjiang-3 (non-cold tolerant) under 4°C treatment. The results will facilitate the discovery of cold resistance genes in asparagus bean, which provides resources for genetic improvement, and shed light on the molecular mechanisms related to cold tolerance in this plant.

## Materials and Methods

### Plant materials

Two asparagus bean cultivars tested in this study, Dubai bean (cold tolerant) and Ningjiang-3(non-cold tolerant), were provided by Mianyang Institute of Agricultural Sciences, Mianyang, Sichuan, China (See S1 Fig). Seeds were presoaked for 4h and incubated in 25°C for 24h, then sowed in pots filled with vermiculite and perlite (V:V = 1:1). Subsequently, these pots were transferred into a growth chamber set to 25°C and 14h light/10h dark period, and watered every two days after seedling emergence. After two weeks, half of the seedlings of each cultivar were transferred to another chamber set to 4°C, while other conditions remained unchanged. 24 hours later, one well developed leaf was harvested from each seedling. Untreated seedlings were used as controls (25°C). More than 20 seedlings were collected for each cultivar. These collected leaves were frozen in liquid nitrogen immediately and stored at −80°C.

### RNA extraction and quality determination

Five leaves harvested from each cultivar were pooled for RNA extraction. Total RNA was extracted with plant RNA Reagent (Invitrogen, Cat.No. 12322–012) following the manufacturer’s protocol. The quality of RNA was determined by a NanoDrop ND-1000 spectrophotometer (Thermo Fisher Scientific, MA, USA). RNA integrity was confirmed by electrophoresis on 1.5% agarose gel.

### cDNA library construction and sequencing

Approximately 20 μg of total RNA from each of four pools (NRT, NCT, CRT and CCT, the first letter “N” stands for the non-cold tolerant cultivar, i.e. Ningjiang-3; “C” represents the cold tolerant cultivar, i.e. Dubai bean; “RT” means room temperature and “CT” indicates cold treatment) was used for Illumina sequencing at Biomarker technologies (Beijing, China). cDNA library construction was performed via following steps. First, mRNA was purified with biotin-Oligo (dT) magnetic bead and was randomly sheared by Fragmentation Buffer. Second, using these sheared fragments as template, first strand cDNA was synthesized with random hexamers, then buffer, dNTPs, RNase H and DNA polymerase I were added to synthesize second-strand cDNA, the product of which was purified with AMPure XP beads. Furthermore, purified double-strand cDNA was end-repaired, poly-A tail-added and ligated to Illumina adapter, then AMPure XP beads were used to select fragment size. Finally, cDNA libraries were obtained by PCR enrichment. In total, we constructed four paired-end libraries and sequencing of the purified libraries were carried out on a Hiseq2500 (Illumina Inc., USA).

### Data analysis

After Illumina sequencing, raw reads were first purified by trimming adapters and removing low-quality sequencing. As a result, high quality reads were obtained, which were called “clean reads”. De novo assembly for clean reads from four libraries were performed collectively by Trinity (release 20131110) [25] to form a single set of non-redundant unigenes. After assembly, unigenes were compared to NR [26], Swiss-Prot [27], GO [28], COG [29], KOG [30] and KEGG [31] databases using BLAST [32] with a cut-off E-value of ≤10^−5^. Moreover, using HMMER software [33] with an E-value threshold of ≤10^−10^, amino acid sequences translated from the unigenes were aligned to Pfam [34] database, in order to gather information about the function and structure of proteins of unigenes.

To get assembly statistics for the percentage of reads that could be mapped back to transcripts (mapped ratio), bowtie (version 1.1.1) [35] was used to align short reads to the transcripts. According to the results, transcript abundance was estimated with RSEM (version 1.2.3) [36]. FPKM (Fragments Per Kilobase of transcript per Million mapped reads) [37] was used to quantify the expression level of unigenes. FPKM/RPKM [38] is currently the most popular method for normalizing RNA-seq gene expression. FPKM is computed similarly to RPKM, except it accounts for the scenario in which only 1 end of a pair-end read is mapped [39]. This value can be directly applied to compare gene expression level among samples. FPKM is calculated as follows:

$$\text{FPKM}=\frac{\text{cDNA Fragments}}{\text{Mapped Fragments}(\text{Millions})\times \text{Transcript Length}(\text{kb})}$$

In this formula, cDNA Fragments is the number of reads that aligned to a specific unigene, Mapped Fragments (Millions) is the total number of reads that aligned to all unigenes, Transcript Length (kb) is the length of the unigene.

### Identification of differential expressed genes (DEGs)

Differentially expressed genes (DEGs) between cold treated and control samples were identified by EBSeq (Version 1.6.0) [40] based on a rigorous algorithm developed by Audic and Claverie [41]. FDR (False Discovery Rate) control method [42] was applied in multiple hypothesis testing to correct the results for p value. An “FDR < 0.01 and FC (fold change)≥2” was set as the threshold to determine the significance of gene expression difference. FC stands for the ratio of FPKM between cold treated and control samples. At last, Mercator tool [43] was employed to analyze these DEGs for functional annotation and classification.

### Validation by qRT-PCR

Leaves harvested from three independent seedlings of both cold-treated and control samples of each cultivar were used as three biological replicates. Total RNA was extracted with RNAiso Plus (TaKaRa, Dalian, China) and cDNA was synthesized by PrimeScript RT reagent Kit With gDNA Eraser (Takara, Dalian, China) according to the manufacturer’s instructions. 16 DEGs, which were up-regulated in both Dubai bean and Ningjiang-3 under cold treatment, were randomly picked out for validation. Primers were designed using Primer3 (http://bioinfo.ut.ee/primer3-0.4.0/) and synthesized by Invitrogen. Details of selected genes and the sequence of primers were listed in S1 Table. All primers were amplified with no template control to make sure the amplicons were not primer dimers. Experiments were carried out with three technical replicates using SYBR® Premix Ex Taq TM II (Takara, Dalian, China) on Bio-Rad CFX96 Real-Time PCR system (Bio-Rad, USA). Gene expression levels were normalized against the geometric mean of two soybean reference genes, *GAPDH* (GenBank: XM_003523083) and *Actin 1* (GenBank: J01298) and calculated by 2^−ΔΔCT^ method.

## Results

### Illumina sequencing, de novo assembly and annotation

In this experiment, we constructed four cDNA libraries, including NCT and NRT, which represent cold-treated and room-temperature (control) samples from Ningjiang-3, respectively, and likewise, CCT and CRT, except that they are from Dubai bean. Then these cDNA libraries were sequenced on Illumina HiSeq2500. After removing sequencing adaptors and low quality data, we obtained 21.54 Gb clean data, more than 90% reads had a quality score of ≥Q30 (sequencing error rate, 0.1%). Statistics of sequencing data is listed in Table 1. All the clean reads were deposited into NCBI Sequence Reads Archive (SRA) with accession number SRP061809.

**Table 1:** Overview of the sequencing results.

Samples	Read Number	Base Number	GC Content	%≥Q30^a^	Mapped Ratio^b^
NCT	18,169,422	4,576,539,390	47.62%	90.49%	86.76%
CCT	20,572,254	5,180,995,901	47.76%	90.16%	86.62%
CRT	23,762,198	5,984,948,631	48.43%	90.49%	86.99%
NRT	23,020,207	5,797,092,468	47.76%	90.44%	86.68%

NCT, Ningjiang-3 (non-cold tolerant) Cold Temperature; NRT, Ningjiang-3 (non-cold tolerant) Room Temperature; CCT, Dubai bean (cold tolerant) Cold Temperature; CRT, Dubai bean (cold tolerant) Room Temperature.

^a^ The percentage of clean reads whose quality score was more than 30.

^b^ The percentage of reads that are mapped to transcripts or unigenes in clean reads.

Transcriptome *de novo* assembly was performed using Trinity, a short reads assembling program [25]. All together, 179,128 transcripts and 88,869 unigenes were generated. The average transcript size exceeded 1237 bp, with the N50 of 2260 bp. The total length of unigene was 56,451,512 bp with a mean length of 635 bp and an N50 of 1169 bp. Detail information is shown in S2 Table and S2 Fig. This Transcriptome Shotgun Assembly project has been deposited at GenBank under the accession GDKT00000000. The version described in this paper is the first version, GDKT01000000.

After assembly, the 88,869 all-unigenes were subjected to public protein databases including NR, Swiss-Prot, GO, COG, KOG and KEGG using BLAST (E value ≤ 10^−5^). Furthermore, using HMMER software (E value ≤ 10^−10^), amino acid sequences translated from the unigenes were aligned to Pfam databse. Eventually, a total of 41,925 (47.18%) unigenes were annotated. Statistics and detailed annotation of the unigenes were presented in S3 and S4 Tables, respectively.

### Gene expression level evaluation

FPKM was used to quantify the expression level of unigenes. The expression level detected by RNA-seq is highly sensitive. Normally, FPKM of genes encoding proteins ranged from 10^−2^ to 10^4^ [44]. Overall distribution of gene expression level of four libraries is shown in S3 Fig, suggesting that the alteration of gene expression is more visible in Dubai bean compared to Ningjiang-3.

### Identification of differential expressed genes (DEGs) under cold stress

In the process of DEGs screening, we used “FDR < 0.01 and FC (fold change)≥2” as the threshold to determine the significance of gene expression difference. FC is the ratio of FPKM between cold treated and control samples. In total, we identified 3510 DEGs in Dubai bean, including 2103 (60%) up-regulated genes and 1407 (40%) down-regulated genes. In Ningjiang-3, we found 2868 DEGs, among which 1786 (62%) were induced and 1082 (38%) were suppressed. Hierarchical cluster analysis was carried out with these DEGs. Genes with same or similar expression profile were clustered, so as to present differential expressing patterns of gene sets under various experimental conditions. Cluster results of DEGs in four libraries are shown in S4 Fig.

1744 DEGs were found in both Dubai bean and Ningjiang-3, reflecting the common cold response in both varieties. When these two cultivars were exposed to low temperature, the number of up-regulated genes was higher than that of down-regulated genes, and Dubai bean had more DEGs than Ningjiang-3, indicating more complex cold response pathways in Dubai bean. The DEGs of Dubai bean and Ningjiang-3 under cold stress are listed in S5 and S6 Tables, respectively.

In general, the change of gene expression in Ningjiang-3 is not as noticeable as Dubai bean. In CCT library, fold change of DEGs ranged from -247 to 657, while in NCT library, this parameter fluctuated between -92 and 263. In CCT and NCT, 46 and 9 DEGs changed at least 100 fold, respectively. In CCT, c50195.graph_c0 was the highest up-regulated gene (654 fold). It was annotated as unknown protein by Mercator tool. Whereas c52324.graph_c0 showed the greatest decrease in expression (-248 fold), which encodes a member of Pectate lyase family protein. In NCT, c49580.graph_c2 exhibited the highest expression level (262 fold) and was also annotated as unknown protein. While c50375.graph_c0 expression displayed the most dramatic repression (-92 fold), which encodes ethylene response factor 1 (ERF1). Since the function of the highest up-regulated gene in each library is unclear, further analysis for functional identification of these genes is needed.

Apart from those genes that showed the greatest changes in expression, some genes with the highest expression level (FPKM value) deserve attention, because these genes may also play important roles in cold tolerance. Genes with the top ten FPKM in CCT and NCT are listed in Table 2. Six genes were common to both libraries, four of which (c34712.graph_c1, c40463.graph_c0, c29440.graph_c0 and c47777.graph_c0) were associated with protein degradation. They encode polyubiquitin UBQ14, UBQ3, matrixin family protein and eukaryotic aspartyl protease family protein, respectively. Another gene, c49271.graph_c0, was related to development, which encodes AtLEA5 (late embryogenesis abundant like protein), also known as SENESCENCE-ASSOCIATED GENE 21 (SAG21). It has a role on oxidative stress tolerance and its mRNA levels are elevated in response to various stresses. Another gene, c45840.graph_c1, had no available description. The expression levels of these six genes were higher in CCT than in NCT.

**Table 2:** List of the top ten genes with the highest FPKM in CCT and NCT. Data in bold symbolize genes shared by CCT and NCT.

Gene ID	Mercator classification	NCT fold (Log_2_FC)	CCT fold (Log_2_FC)
c49258.graph_c0	not assigned.unknown	-	3.09
c49297.graph_c0	RNA.regulation of transcription	-	3.31
c49349.graph_c0	RNA.regulation of transcription	-	4.37
c29841.graph_c0	RNA.regulation of transcription	-	4.33
c34712.graph_c1	protein.degradation	2.47	2.95
c40463.graph_c0	protein.degradation	2.57	3.67
c29440.graph_c0	protein.degradation	3.36	4.07
c49271.graph_c0	development.late embryogenesis abundant	5.41	5.53
c47777.graph_c0	protein.degradation	5.53	6.10
c45840.graph_c1	not assigned.unknown	3.09	4.02
c48464.graph_c0	protein.degradation	2.06	-
c49265.graph_c0	stress.abiotic	2.38	-
c49294.graph_c0	hormone metabolism.gibberelin.	2.47	-
c49308.graph_c0	polyamine metabolism.synthesis	2.32	-

Besides, CCT and NCT owned four unique genes, respectively. In CCT, three of four specific genes (c49297.graph_c0, c49349.graph_c0 and c29841.graph_c0) were related to regulation of transcription, which encode related to ABI3/VP1 2 (RAV2), salt tolerance zinc finger (STZ), and zinc finger (CCCH-type) family protein, respectively. The other gene c49258.graph_c0 had unknown function. In NCT, the four exclusive genes (c48464.graph_c0, c49265.graph_c0, c49294.graph_c0 and c49308.graph_c0) encode ubiquitin 11 (UBQ11), germin-like protein (GLP1), Gibberellin-regulated family protein and S-adenosylmethionine decarboxylase proenzyme, respectively.

### DEG classification

**Fig 1:**
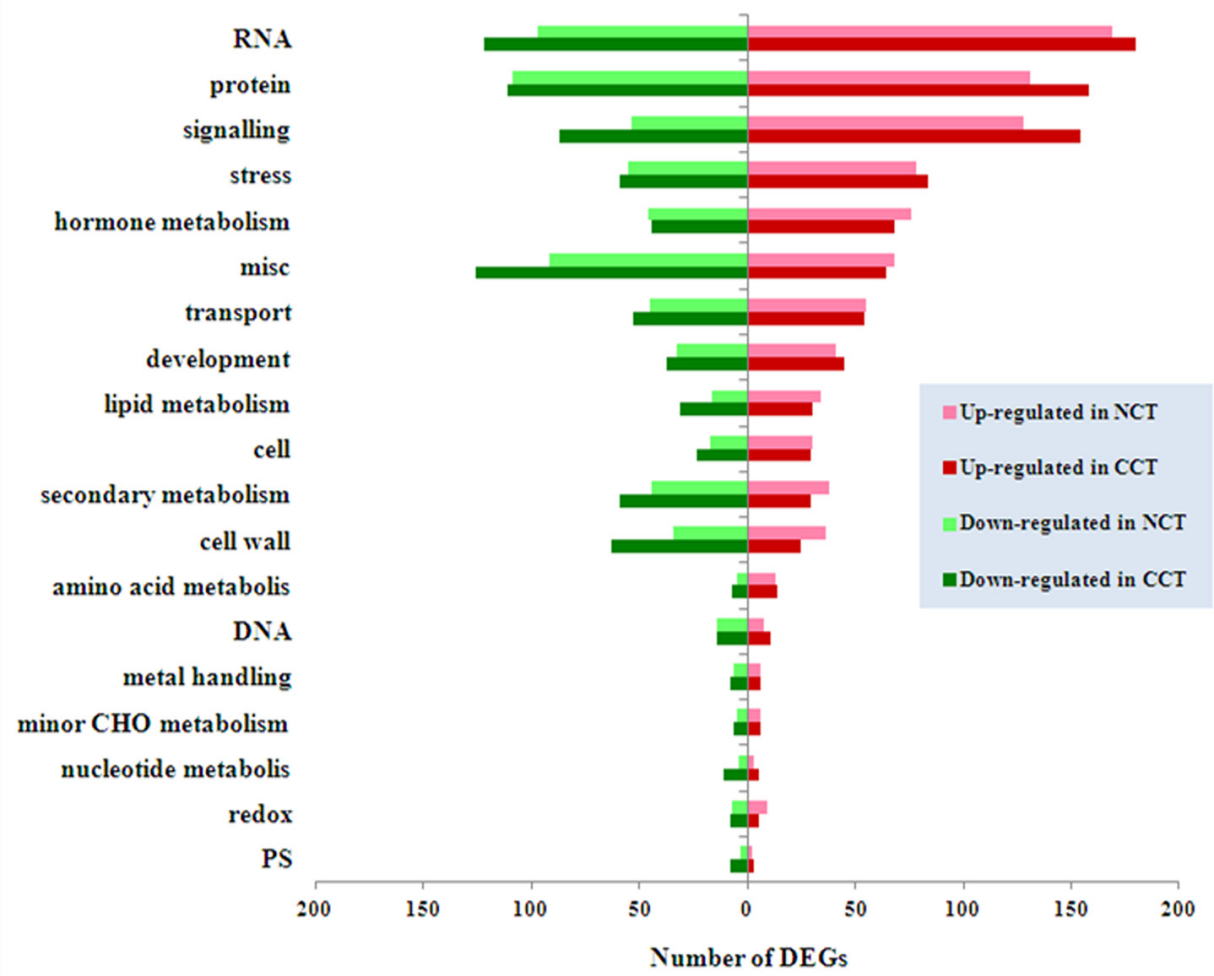
Functional classification of DEGs in NCT and CCT.

In order to better understand the biological function of DEGs, we used Mercator pipeline to annotate and cluster DEGs of CCT and NCT libraries, annotation results of which are listed in S7 and S8 Tables, respectively. Clusters with more than ten DEGs are displayed in Fig 1. The results showed that in both libraries, genes involved in RNA comprised the largest functional group. Other major groups included protein, signaling, stress and hormone metabolism. Therefore, our analysis is focused on these aspects. In these groups, the number of up-regulated or down-regulated genes in CCT was higher than NCT, except for hormone metabolism group. In CCT and NCT, 1774 (50%) and 1358 (47%) DEGs were clustered in “not assigned”, respectively. Some of them may be novel genes involving in cold response that have never been reported.

### Transcription factors (TFs)

Transcriptional control of the expression of stress-responsive genes is a crucial part of the plant response to various abiotic and biotic stresses [45]. In CCT and NCT, RNA was the biggest group in Mercator classification, in which “regulation of transcription” was the highest proportion. A total of 33 TF families were identified in DEGs of both libraries (Table 3). The majority of TFs belonged to AP2/EREBP, WRKY, MYB, zinc finger family (including C2C2, C2H2, and C3H), bHLH and NAC families. In most TF families, the number of up-regulated DEGs exceeded down-regulated DEGs. However, in a few TF families like HB (Homobox), there were more down-regulated DEGs. TFs in JUMONJI, Psudo ARR, PHD, ELF3, AtSR and ARF families were all up-regulated. c37420.graph_c0 encodes ARF (Auxin responsive factor) family TF and was up-regulated by 4.1 fold in NCT, but it was not differentially expressed (not a DEG) in CCT. On the other hand, TFs in Zn-finger (CCHC), GeBP, TCP, zf-HD families were all down-regulated. So many TFs were regulated by low temperature, implying the complexity of cold regulation network in asparagus bean.

**Table 3:** Differential expressed transcription factors in CCT and NCT.

	Up-regulated TF		Down-regulated TF	
TF	CCT	NCT	CCT	NCT
AP2/EREBP	28	14	7	13
WRKY	22	26	2	2
MYB	15	14	8	6
C2H2	13	12	3	5
C2C2	11	9	6	6
NAC	8	6	1	0
bHLH	7	11	14	2
PHOR1	6	5	2	0
GRAS	6	4	4	2
bZIP	5	5	5	4
HSF	4	2	2	1
MYB-related	4	6	6	4
JUMONJI	3	3	0	0
GARP	3	4	1	2
Psudo ARR	2	1	0	0
Trihelix	2	2	1	2
ABI3/VP1	2	2	1	1
C3H	2	1	2	1
AS2	2	3	3	2
PHD	1	1	0	0
ELF3	1	1	0	0
AtSR	1	2	0	0
FHA	1	1	1	1
Aux/IAA	1	3	1	0
OFP	1	2	3	1
ARR	1	2	2	1
HB	1	1	4	6
ARF	0	1	0	0
Zn-finger(CCHC)	0	0	1	1
GeBP	0	0	1	2
TCP	0	0	2	1
CCAAT box	0	4	4	2
ZF-HD	0	0	6	2
Total	153	148	93	70

**Fig 2:**
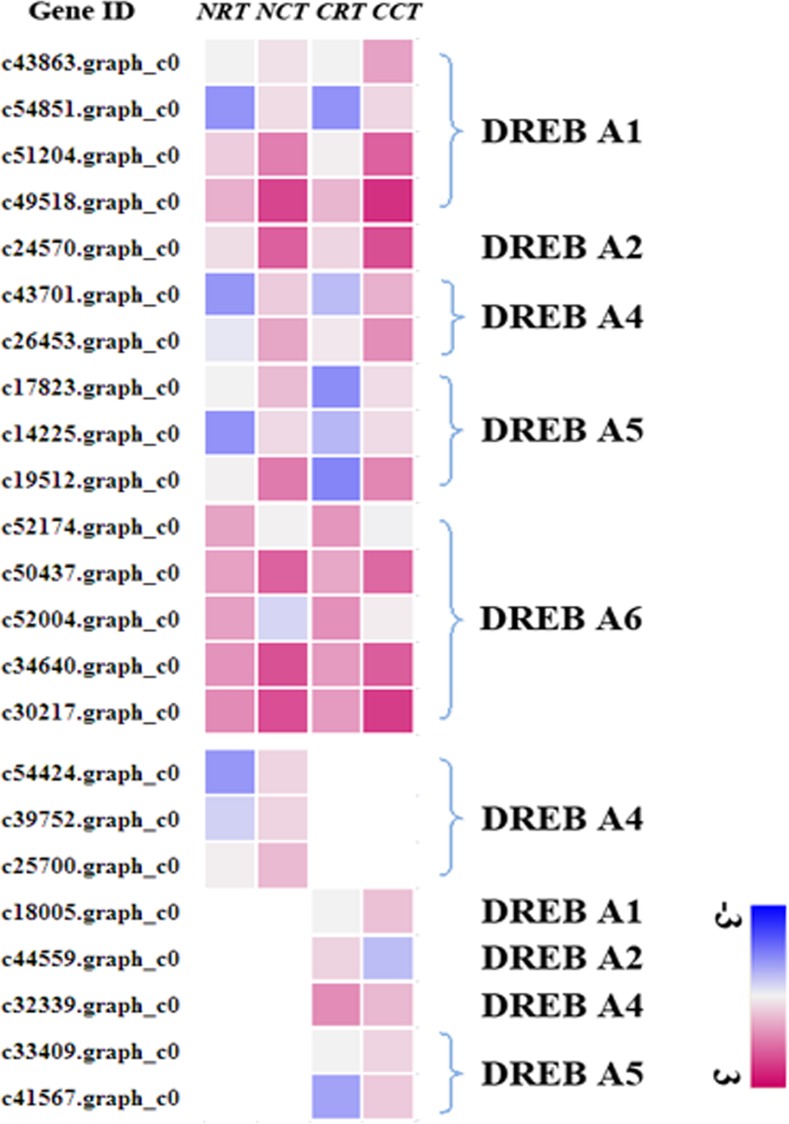
Expression level of TFs belonged to DREB family in four libraries. Color bins represent Log_10_FPKM of a gene.

Among all the TF families that have been identified, AP2/ERF (also called AP2/EREBP) family is the largest. AP2/ERF transcription factors have been implicated in hormone, sugar and redox signaling in context of abiotic stresses such as cold and drought [46]. DREB (dehydration responsive element binding protein) is a subfamily belonged to AP2/ERF. This group of subfamily was further divided into six subgroups (A-1 to A-6) [1]. DREB1/CBF genes belong to the A1 group and are quickly and transiently induced by low temperature, and their products activate the expression of multiple stress-inducible target genes. While the DREB2 genes, which belong to the A2 group, are induced by dehydration, leading to the expression of drought-responsive genes [47]. In CCT and NCT, there were 15 common TFs belonged to DREB (Fig 2), including A1, A2, A4, A5 and A6 subgroup. They exhibited the same expression pattern and similar expression level. Most of these TFs were up-regulated, whereas two TFs, c52174.graph_c0 and c52004.graph_c0, were down-regulated, which belonged to A6. The expression level of TFs in A1, A2 and A4 group were higher in CCT than NCT. On the contrary, TFs in A5 group expressed more in NCT.

Additionally, there were three unique DREB TFs in NCT, which were all up-regulated and belonged to A4 group. Also, five DREB TFs were CCT-specific. Of these, c18005.graph_c0 belonged to A1 and its expression increased up to 33-fold under cold stress. c44559.graph_c0 and c32339.graph_c0 belonged to A2 and A4, respectively, which were both down-regulated. The other two genes belonged to A5 and were both up-regulated.

### Protein, signaling, stress and hormone metabolism

**Fig 3:**
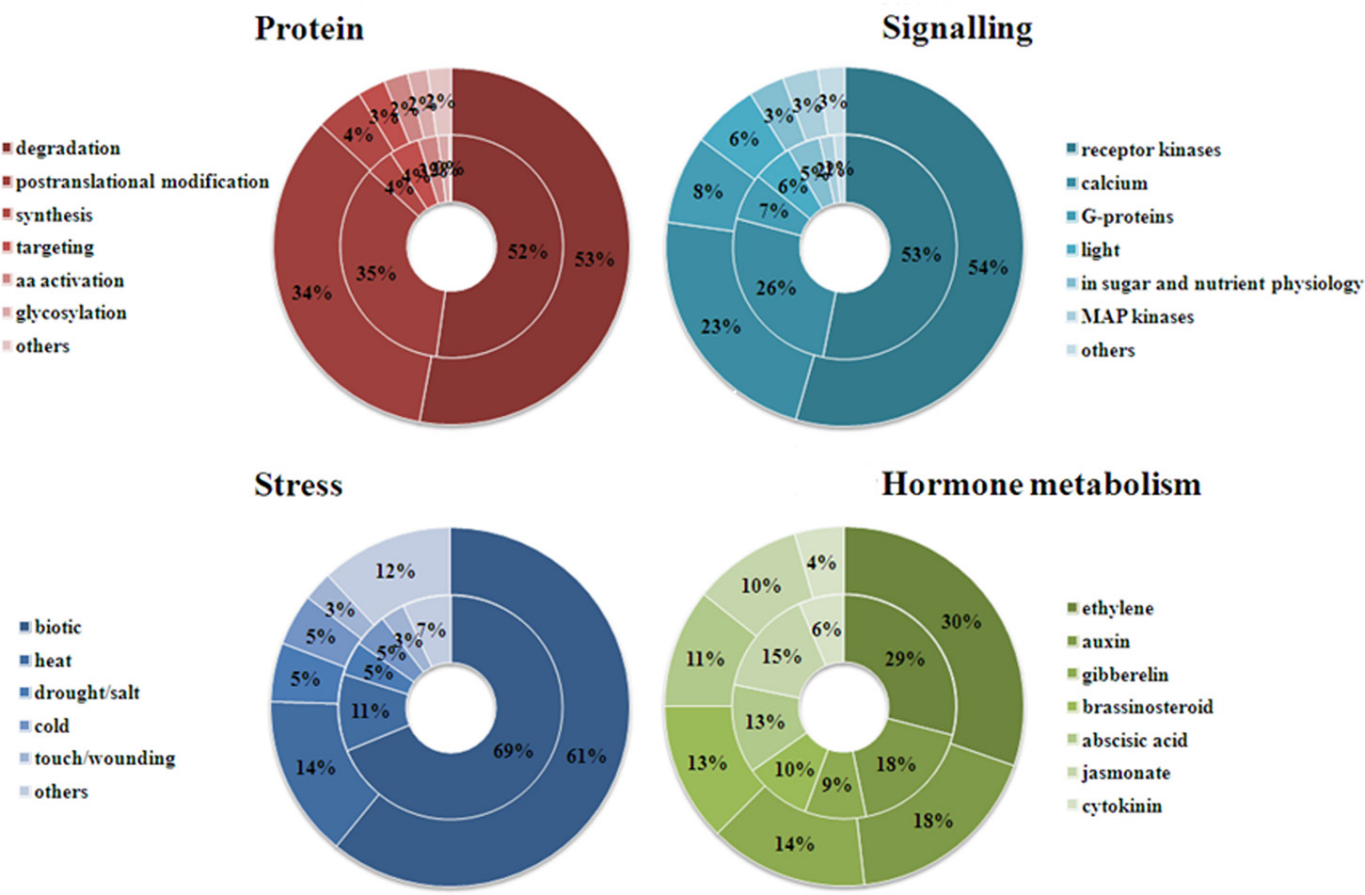
Four major groups of DEG classification in CCT and NCT. The outer circle represents CCT, while the inner circle symbolizes NCT. Each group is further divided into several subgroups, the percentage of which is shown.

Aside from RNA, DEGs in each of the other four major groups were further clustered in smaller subgroups (Fig 3). In protein group, “protein degradation” was the biggest subgroup. There were146 and 130 DEGs related to protein degradation in CCT and NCT, respectively, 87 and 89 of which involved in ubiquitin pathway. The second largest subgroup was “posttranslational modification”. There were 94 and 86 DEGs involved in this subgroup in CCT and NCT, respectively, which mainly encode protein kinases and protein phosphatase. Protein phosphorylation/dephosphorylation is now well demonstrated to play a part in signal transduction during cold acclimation.

In signaling group, “receptor kinases” was the largest subgroup, which accounted for 54% and 53% in CCT and NCT, respectively. The next biggest subgroup was “calcium”, which accounted for 23% and 26% in CCT and NCT, respectively. There is mounting evidence that calcium is an important second messenger in a low temperature signal transduction pathway involved in regulating the cold-acclimation response [2]. DEGs in this group primarily encode Calcium-binding EF-hand family protein and Calmodulin-binding protein.

In stress group, “biotic” stress was the largest subgroup, which accounted for 61% and 64% in CCT and NCT, respectively. And in this subgroup, many DEGs were related to PR (pathogen-related) protein, suggesting that some disease resistant genes were also induced by low temperature. The other subgroups belonged to abiotic stresses including “heat”, “drought/salt”, “cold”, and “touch/wounding”. Some genes related to a range of stress conditions were also induced by low temperature, indicating a cross-talk between biotic and abiotic stress and between different abiotic stresses. DEGs related to “heat” mainly encode chaperone dnaJ-domain superfamily protein, heat shock protein and heat shock factor.

Plant hormones play critical roles in plants’ adaption to changing environments, by mediating growth, development, nutrient allocation, and source/sink transitions [48]. During cold stress, the expression of genes related to biosynthesis and metabolism of phytohormones like IAA, GA, ABA, CK, ETH and BR have also been influenced. In hormone metabolism group, “ethylene” was the biggest subgroup. DEGs in this subgroup mainly encode ACC synthase and ethylene response factor. The second largest subgroup was “auxin”. DEGs in this subgroup principally encode SAUR-like auxin-responsive protein family and auxin-responsive family protein. Since cold-regulated gene expression itself involves multiple mechanisms including both “ABA-dependent” and “ABA-independent” pathways [2], ABA plays a crucial role in cold tolerance. DEGs in subgroup “ABA” primarily encode nine-cis-epoxycarotenoid dioxygenase (NCED), ABA 8'-hydroxylase, highly ABA-induced PP2C gene 3 (HAI3), GRAM domain-containing protein. Noteworthy, two genes, c42395.graph_c0 and c52708.graph_c0, encoding ABRE binding factor 4 (ABF4) were down-regulated by 3.5 and 5.6 fold in NCT, respectively. Another two genes, c53208.graph_c0 and c53371.graph_c0, encoding ABRE binding protein 3 (AREB3) were down-regulated by 4.9 and 7.8 fold in NCT, respectively. ABA-responsive element (ABRE) is the major cis-element for ABA-responsive gene expression under osmotic stress conditions, and ABRE binding protein and ABRE-binding factor TFs control these genes’ expression in an ABA-dependent manner [49]. However, these four genes were not differentially expressed in CCT.

### qRT-PCR validation

**Fig 4:**
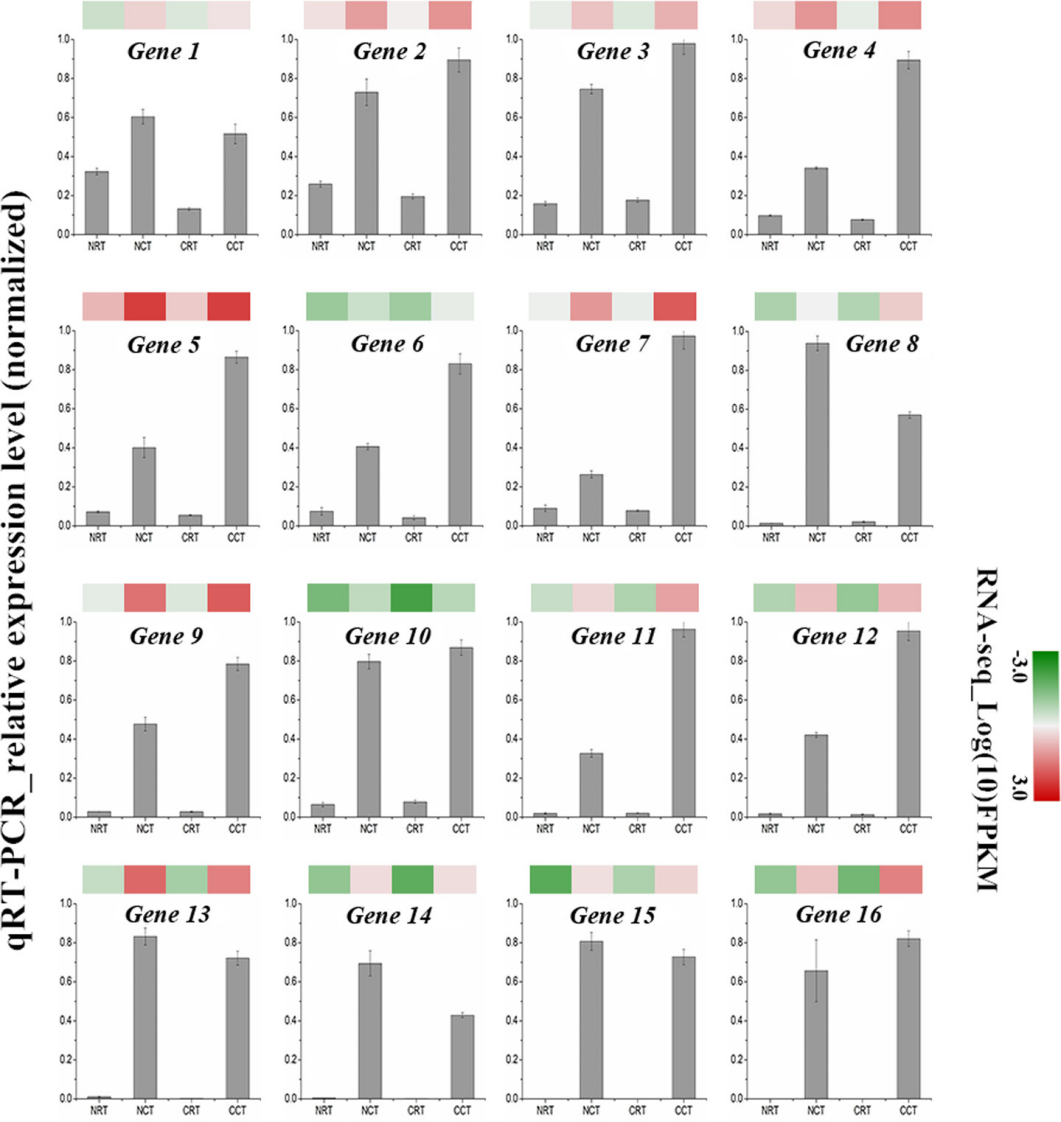
Expression pattern of 16 selected genes as obtained by RNA-seq and qRT-PCR. The gray bars represent the relative expression level of one gene after normalized to the geometric mean of two soybean reference genes. Color panels above the bars symbolize log_10_FPKM of this gene as measured by RNA-seq.

**Fig 5:**
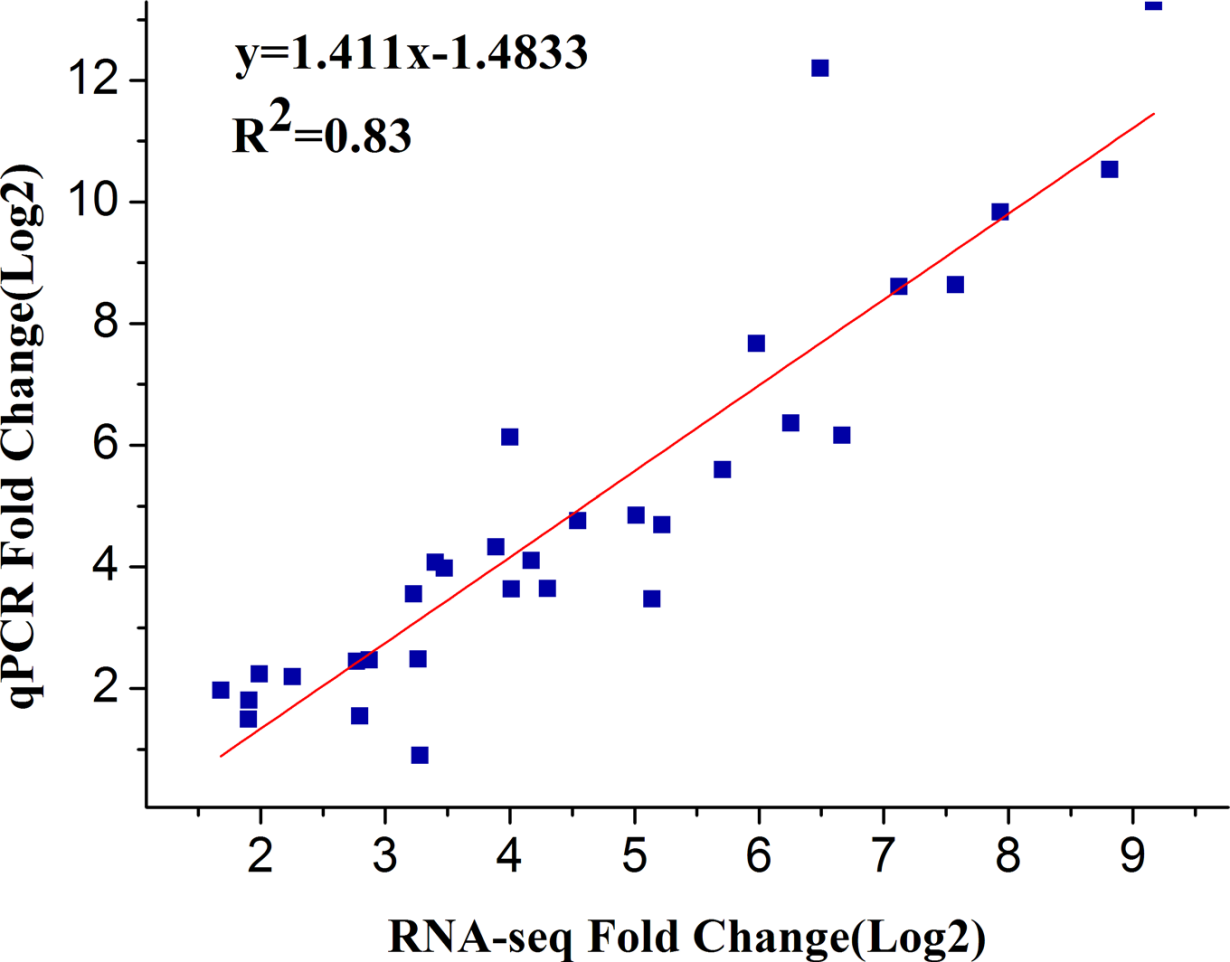
Scatter plot of 16 selected genes based on fold change measured by RNA-seq and by qRT-PCR analysis. A linear trend line is shown.

To verify the reliability and accuracy of our transcriptome data, we randomly selected 16 up-regulated unigenes from common DEGs in CCT and NCT libraries and evaluated their expression profiles using quantitative real-time PCR. Two most widely used reference genes, *actin* and *GAPDH*, were selected for internal controls. The expression patterns of selected genes were determined and further compared with those of in RNA-seq assay. Nearly all of these genes displayed similar expression trend in both techniques (Fig 4). Moreover, the correlation between qRT-PCR and RNA-seq was measured by scatter plotting log2-fold changes (Fig 5), which showed a positive correlation coefficient (Pearson coefficient R^2^ = 0.83).

## Discussion

### Up-regulated genes and down-regulated genes

After 24h incubation at 4°C, the number of up-regulated genes clearly exceeded that of down-regulated genes in both CCT and NCT libraries. There is a notion that cold-stress-induced genes are necessary for cold tolerance whereas cold-repressed genes are important for active growth [50]. Some reported researches of *Arabidopsis* cold-responsive transcriptome showed that the number of up-regulated genes was more than that of down-regulated genes [51–54]. *Arabidopsis* is capable of cold acclimation, whereas asparagus bean is not able to cold acclimate. When exposed to low temperature, they both possess more up-regulated genes. On that account, it appears that the ability of cold acclimation is not the key point to determine whether up-regulated genes are more or less. Besides, similar results have been described in transciptome research of barley [55], cassava [56], *Camellia sinensis* [57] and *Vitis amurensis* [23, 58]. However, in *Anthurium* [59], *Ammopiptanthus mongolicus* [60] and *Populus simonii* [61], more down-regulated genes were found. Taken together, the relative ratio between up-regulated genes and down-regulated genes in response to cold stress may vary with different plant species.

### Expression profile of DREB1/CBF

One of the major advances in the past two decades of cold tolerance research was the discovery of the cold-inducible CBF or DREB1 (C-repeat-binding/dehydration-responsive element) transcriptional activators [5]. The transcriptional factor DREB1/CBF specifically interacts with the dehydration-responsive element (DRE)/C-repeat (CRT) *cis*-acting element (A/GCCGAC) and controls the expression of many stress-inducible genes in *Arabidopsis* [62]. There are 6 members in this subfamily including CBF1, CBF2, CBF3, CBF4, DDF1 and DDF2 [63]. As shown in Fig 2, four TFs belonged to DREB1 are shared by CCT and NCT. Of these, c43863.graph_c0 encodes DDF2 (DWARF AND DELAYED FLOWERING 2). DDF2 is the closest homolog of DDF1, and both are induced by high-salinity stress [64]. Overexpression of DDF1 or DDF2 reduces bioactive GAs in *Arabidopsis* [65]. The reduction of bio-active GAs leads to accumulation of the nuclear-localized growth-repressing DELLA proteins [66]. It has been proposed that DELLAs permit flexible and appropriate modulation of plant growth in response to environmental changes [67]. This gene was inductive, i.e. it was not expressed under normal conditions but was induced by exposure to low temperature. In CCT and NCT, it was up-regulated by 74 fold and 13 fold, respectively. Another two genes (c54851.graph_c0 and c51204.graph_c0) encode CBF4. In CCT they were up-regulated by 12 fold and 51 fold, respectively, while in NCT they were up-regulated by 11 fold and 10 fold, separately. Transcription factor CBF4 is a regulator of drought adaptation in *Arabidopsis* [68]. Unlike CBF1, CBF2 and CBF3, which are known to be induced in cold stress, CBF4 gene expression is up-regulated by drought stress, but not by low temperature. Overexpression of CBF4 in transgenic *Arabidopsis* plants can activate CRT/DRE containing downstream genes, thus making the transgenic plants more tolerant to freezing and drought stress. Another gene, c49518.graph_c0 encodes CBF3/DREB1A, which was up-regulated by 47 fold and 25 fold in CCT and NCT, respectively. This gene is involved in response to low temperature and abscisic acid. Liu et al. [69] have shown that overexpression of CBF3/DREB1A in transgenic *Arabidopsis* plants causes constitutive expression of *RD29A* and enhances both the freezing and drought tolerance of the transgenic plants. A major positive regulator of CBF3 is ICE1 (inducer of CBF expression1), a basic helix-loop-helix transcription factor that binds to multiple Myc DNA regulatory elements present in the CBF3 promoter and stimulates CBF3 transcription [70]. In our RNA-seq results, we also found a gene, c25032.graph_c0, which encodes ICE1. It was expressed in both cold treated and control samples, but it was not a differentially expressed gene. This is consistent with its constitutively expressed property [71]. Therefore, the ICE1-CBF3-COR transcription cascade may also exist in asparagus bean.

Noticeably, there was a unique gene in CCT, c18005.graph_c0, which encodes CBF1. In control samples, its expression level was zero. But in low temperature, it was up-regulated by 33 fold. Increased expression of *Arabidopsis* CBF1 induced *COR* gene expression and increased the freezing tolerance of non-acclimated *Arabidopsis* plants [72]. Moreover, Achard et al. [66] found that transgenic plants that constitutively express *CBF1* accumulate less bioactive GA, leading to the accumulation of DELLA protein, and as a consequence exhibit dwarfism, late flowering and enhanced freezing tolerance.

### Possible mechanism underlying cold tolerance of Dubai bean

Based on our transcriptome data, we speculate that there may exist three reasons that contributed to the higher cold tolerance of Dubai bean. Firstly, during cold stress, some previously reported genes responsible for cold tolerance were differentially expressed in Dubai bean, but not in Ningjiang-3. An example is c41219.graph_c0. It encodes ESK1, which functions as a negative regulator of cold acclimation [73]. Mutation in the *ESK1* gene provides strong freezing tolerance. Unlike CBF pathway, the *ESK1* gene may participate in the control of another set of freezing tolerance responses that includes synthesis of proline and sugars and expression of RAB18 [2]. This gene was down-regulated by 5.4 fold only in CCT, which agrees with its negative regulator role.

In the second place, Dubai bean featured higher expression level of some commonly changed genes. For instance, in “heat” of stress group, three genes (c24017.graph_c0, c50903.graph_c0 and c51953.graph_c0) encode heat shock factor (HSF). They were all up-regulated in both libraries, but in CCT its expression level was higher than NCT. In *Arabidopsis*, four HSF were also found to be cold responsive and up-regulated [52]. HSF regulate the expression of heat shock protein (HSP), which is also induced by low temperature in plants. These HSPs function in membrane protection, in the refolding of denatured proteins and in preventing protein aggregation [74].

Thirdly, the results of Mercator classification showed that in most functional groups, the number of DEGs was larger in CCT compared to NCT (Fig 1), implying that Dubai bean has more complex regulatory networks to deal with cold stress. For example, Mitogen activated protein kinase (MAPKs) are mediators of several signal transduction pathways in eukaryotic cells, including responses to a variety of environmental stresses [7]. Transcripts for a MAPK kinase kinase, ATMEKK1, and a MAPK, ATMPK3 have been shown to accumulate rapidly in *Arabidopsis* in response to low temperature [75]. In “MAP kinases” of signaling group, there were eight genes in CCT, while the number was four in NCT.

## Conclusion

In this experiment, we analyzed the global transcriptome modification of two asparagus bean cultivars, Dubai bean and Ningjiang-3, that are differing in cold tolerance under low temperature. 3510 and 2868 DEGs were identified from two cultivars, respectively, and functional analysis with these DEGs was performed. The results showed that the most prevalent functional groups in both genotypes were RNA, protein, signaling, stress and hormone metabolism. We further analyzed these groups separately. TFs were the largest subgroup in RNA group. We focused on the expression pattern of TFs that belonged to DREB family, especially DREB A1 in both cultivars, which were found to be expressed with higher level in CCT library. Moreover, there was a unique gene encoding CBF1 existed in CCT. We also discussed the possible reasons why Dubai bean is more tolerant to cold stress than Ningjiang-3, including higher expression level of cold responsive genes and more abundant DEGs in most of common functional groups, highlighting the multiple gene control and complexity of cold stress response mechanism in Dubai bean. In summary, as a non-cold acclimated crop, asparagus bean also has CBF pathway, which may play an important role in cold tolerance of Dubai bean.

## Supporting Information

S1 Fig Dubai bean (left) and Ningjiang-3 (right). This picture is taken in mid-October at Mianyang Institute of Agricultural Sciences, Sichuan, China.
(TIF)

S2 Fig The length distribution of unigenes. (TIF)

S3 Fig Overall distribution of unigenes in four libraries measured by FPKM. (TIF)

S4 Fig Hierarchical cluster analysis of DEGs in four libraries. (TIF)

S1 Table Details of selected genes and the sequence of primers. Functional annotation of these genes was performed by Mercator pipeline.
(XLSX)

S2 Table Statistics of de novo assembly results. (DOCX)

S3 Table Statistics of unigene annotation. All unigene sequences were subjected to a BLAST comparison against NR, Swiss-Prot, GO, COG, KOG, KEGG and Pfam databases.
(DOCX)

S4 Table Functional annotation of the unigenes. (XLS)

S5 Table The DEGs of Dubai bean during cold stress. Unigenes that expressed differentially during chilling tolerance were list in this file.
(XLS)

S6 Table The DEGs of Ningjiang-3 under 4°C treatment. (XLS)

S7 Table Functional annotation of DEGs of Dubai bean by Mercator pipeline. (XLSX)

S8 Table Functional annotation of DEGs of Ningjiang-3 by Mercator pipeline. (XLSX)
