# Supplementary figures and images for: Transcriptome Profiling of Two Asparagus Bean (Vigna unguiculata subsp. sesquipedalis) Cultivars Differing in Chilling Tolerance under Cold Stress
Source: PLoS One. 2016 Mar 8;11(3):e0151105. doi: 10.1371/journal.pone.0151105 (PMC4783050; doi:10.1371/journal.pone.0151105)

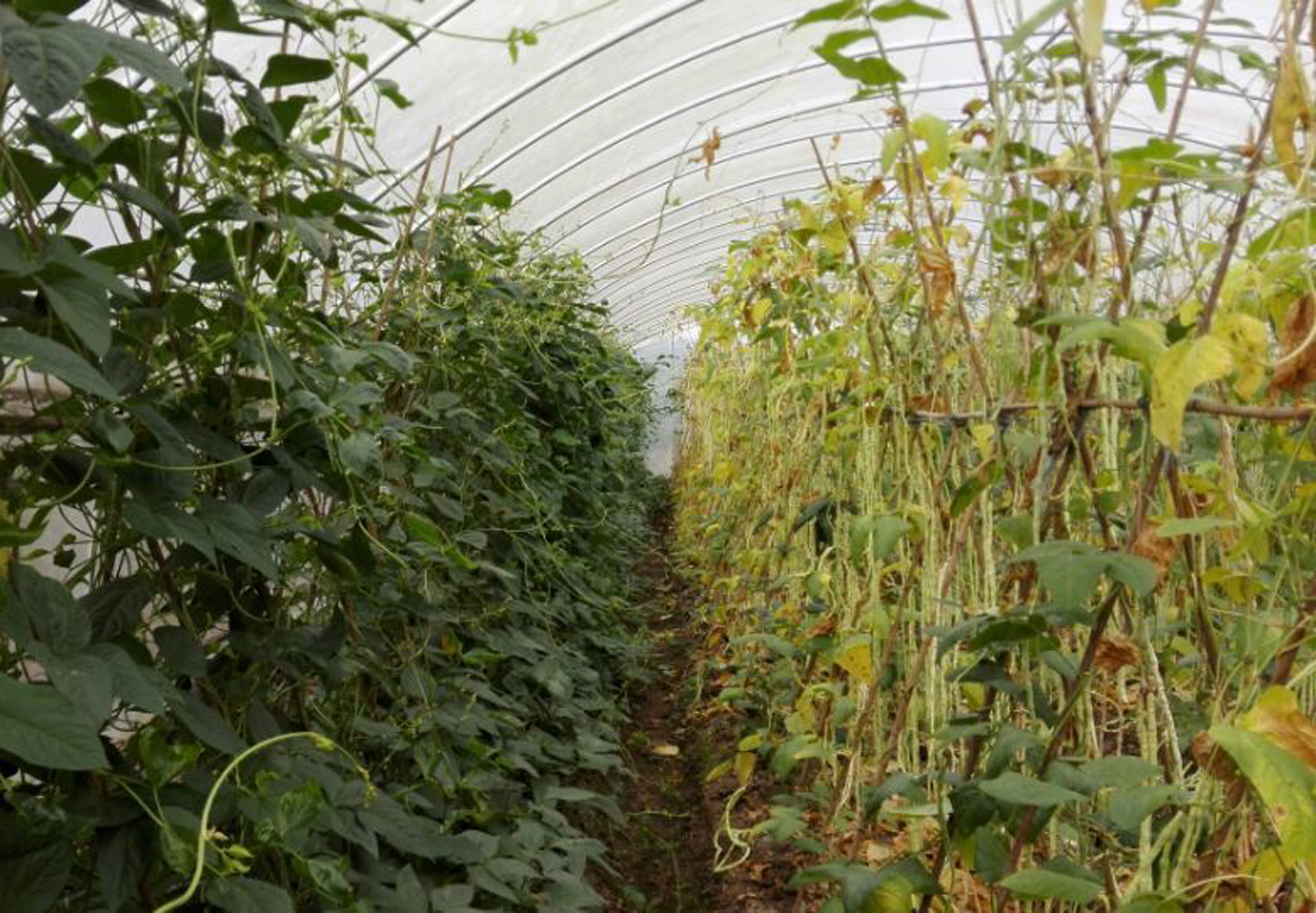

Supplement: S1 Fig — This picture is taken in mid-October at Mianyang Institute of Agricultural Sciences, Sichuan, China. (TIF) [file pone.0151105.s001.tif]

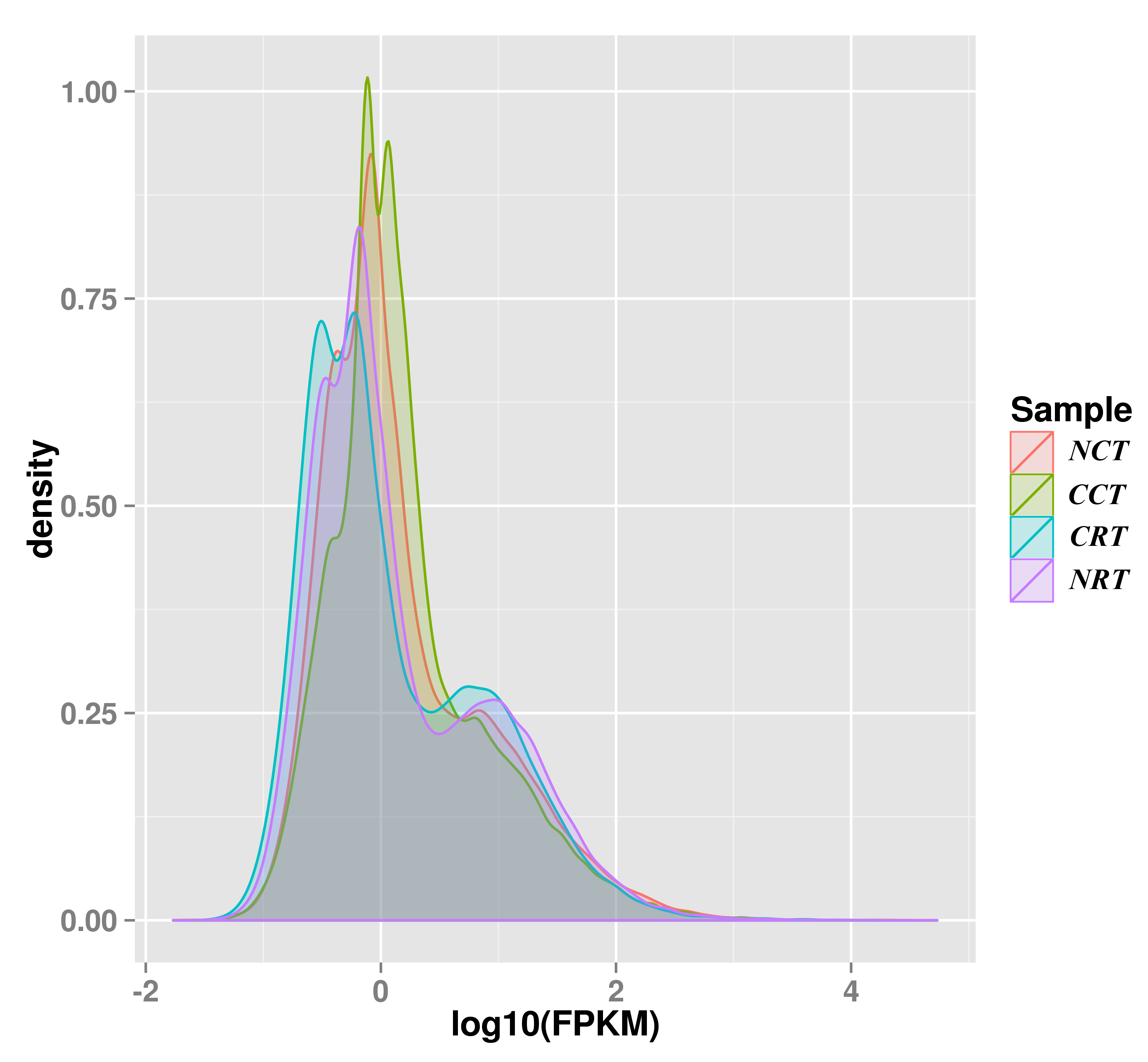

Supplement: S2 Fig — (TIF) [file pone.0151105.s002.tif]

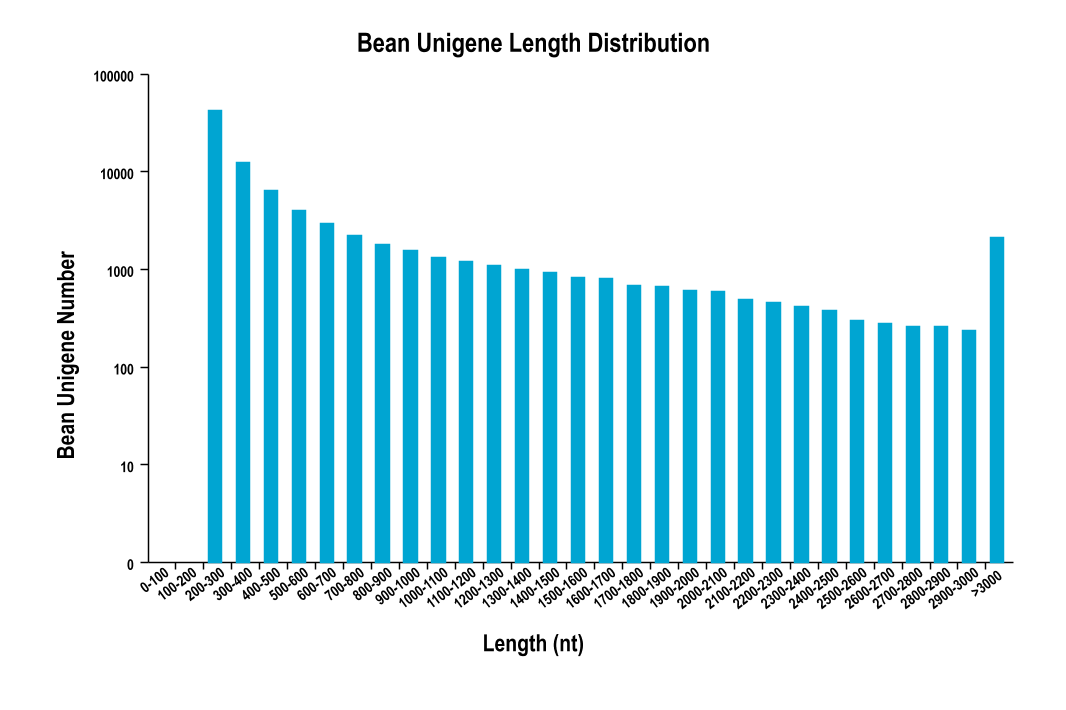

Supplement: S3 Fig — (TIF) [file pone.0151105.s003.tif]

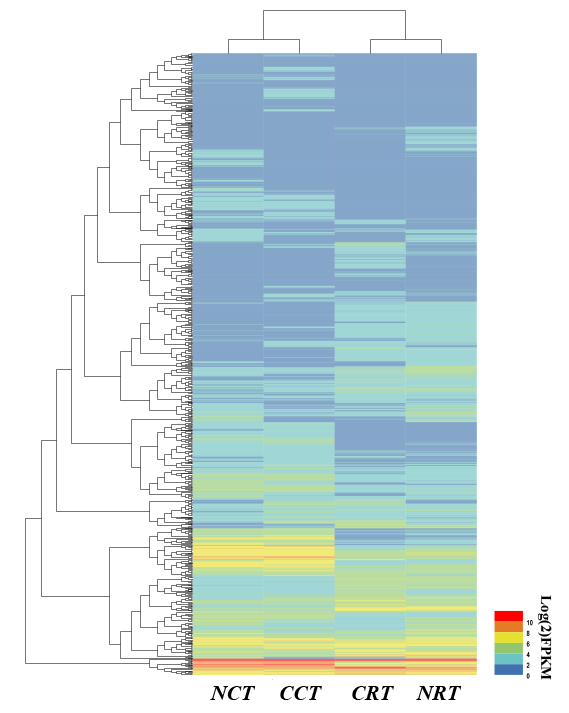

Supplement: S4 Fig — (TIF) [file pone.0151105.s004.tif]
