# Supplementary material for: Transcriptome Profiling of Two Asparagus Bean (Vigna unguiculata subsp. sesquipedalis) Cultivars Differing in Chilling Tolerance under Cold Stress
Source: PLoS One. 2016 Mar 8;11(3):e0151105. doi: 10.1371/journal.pone.0151105 (PMC4783050; doi:10.1371/journal.pone.0151105)
Supplement: S2 Table — (DOCX) [file pone.0151105.s006.docx]

**Table S2. Statistics of assembly results.**

| **Length Range** | **Contig** | **Transcript** | **Unigene** |
| --- | --- | --- | --- |
| 200-300 | 7,316,652(99.27%)* | 48,104(26.85%) | 42,437(47.75%) |
| 300-500 | 22,711(0.31%) | 26,686(14.90%) | 18,983(21.36%) |
| 500-1000 | 15,239(0.21%) | 28,041(15.65%) | 12,504(14.07%) |
| 1000-2000 | 10,289(0.14%) | 36,711(20.49%) | 9,170(10.32%) |
| 2000+ | 5,631(0.08%) | 39,586(22.10%) | 5,775(6.50%) |
| Total Number | 7,370,522 | 179,128 | 88,869 |
| Total Length | 350,521,826 | 221,695,829 | 56,451,512 |
| N50 Length | 47 | 2,260 | 1,169 |
| Mean Length | 47.56 | 1237.64 | 635.22 |
