# Supplementary material for: Transcriptome Profiling of Two Asparagus Bean (Vigna unguiculata subsp. sesquipedalis) Cultivars Differing in Chilling Tolerance under Cold Stress
Source: PLoS One. 2016 Mar 8;11(3):e0151105. doi: 10.1371/journal.pone.0151105 (PMC4783050; doi:10.1371/journal.pone.0151105)
Supplement: S3 Table — All unigene sequences were subjected to a BLAST comparison against NR, Swiss-Prot, GO, COG, KOG, KEGG and Pfam databases. (DOCX) [file pone.0151105.s007.docx]

**Table S3. Statistics of unigene annotation.**

| **Annotated databases** | **Unigene** | **≥300nt** | **≥1000nt** |
| --- | --- | --- | --- |
| COG | 14,027 | 9,657 | 5,765 |
| GO | 19,167 | 13,250 | 6,744 |
| KEGG | 8,780 | 6,385 | 3,377 |
| KOG | 23,193 | 16,628 | 8,555 |
| Pfam | 25,764 | 19,956 | 11,743 |
| Swiss-Prot | 25,145 | 19,476 | 10,325 |
| nr | 39,467 | 28,361 | 13,941 |
| All | 41,925 | 28,817 | 13,954 |
